# Head-to-Head Comparison of Fibroblast Activation Protein Inhibitors (FAPI) Radiotracers versus [^18^F]F-FDG in Oncology: A Systematic Review

**DOI:** 10.3390/ijms222011192

**Published:** 2021-10-17

**Authors:** Giorgio Treglia, Barbara Muoio, Hessamoddin Roustaei, Zahra Kiamanesh, Kamran Aryana, Ramin Sadeghi

**Affiliations:** 1 Clinic of Nuclear Medicine, Imaging Institute of Southern Switzerland, Ente Ospedaliero Cantonale, 6500 Bellinzona, Switzerland; 2 Department of Nuclear Medicine and Molecular Imaging, Lausanne University Hospital, 1011 Lausanne, Switzerland; 3 Academic Education, Research and Innovation Area, General Directorate, Ente Ospedaliero Cantonale, 6500 Bellinzona, Switzerland; 4 Faculty of Biology and Medicine, University of Lausanne, 1011 Lausanne, Switzerland; 5 Faculty of Biomedical Sciences, Università della Svizzera italiana, 6900 Lugano, Switzerland; 6 Department of Medicine and Oncology, Institute of Southern Switzerland, Ente Ospedaliero Cantonale, 6500 Bellinzona, Switzerland; barbara.muoio@eoc.ch; 7 Nuclear Medicine Research Center, Mashhad University of Medical Sciences, 9919991766 Mashhad, Iran; roustaeifh981@mums.ac.ir (H.R.); kiamaneshz@gmail.com (Z.K.); aryanak@mums.ac.ir (K.A.); sadeghir@mums.ac.ir (R.S.)

**Keywords:** PET, positron emission tomography, FAPI, fibroblast activation protein, fluorodeoxyglucose, FDG, oncology, cancer, imaging, systematic review

## Abstract

Several recent studies comparing radiolabeled fibroblast activation protein inhibitors (FAPI) and fluorine-18 fluorodeoxyglucose ([^18^F]F-FDG) as positron emission tomography (PET) radiotracers in oncology have been published. The aim of this systematic review is to perform an updated evidence-based summary about the comparison of these PET radiotracers in oncology to better address further research in this setting. Studies or subsets of studies comparing radiolabeled FAPI and [^18^F]F-FDG as PET radiotracers in oncology were eligible for inclusion in this systematic review. A systematic literature search of PubMed/MEDLINE and Cochrane library databases was performed until August 2021. Literature data about the comparison of [^18^F]F-FDG and radiolabeled FAPI are rapidly increasing. Overall, taking into account radiotracer uptake and tumor-to-background uptake ratio, compared to [^18^F]F-FDG PET, an equal or higher detection of primary tumors and/or metastatic lesions was usually demonstrated by using radiolabeled FAPI PET. In particular, the cancer entities with better detection rate of tumor lesions by using radiolabeled FAPI PET, compared to [^18^F]F-FDG PET, were gastrointestinal tumors, liver tumors, breast cancer and nasopharyngeal carcinoma. Further comparison studies are needed to better evaluate the best field of application of radiolabeled FAPI PET.

## 1. Introduction

Positron emission tomography (PET) is a functional imaging technique extensively used in oncology to diagnose tumors early, even in the absence of morphological abnormalities. Hybrid imaging modalities, including PET/computed tomography (PET/CT) and PET/magnetic resonance imaging (PET/MRI), are currently available and may allow to combine functional and morphological information on cancer patients. Different PET radiotracers evaluating different metabolic pathways or receptor statuses may be used in this setting [1,2,3,4]. Although many PET radiotracers are currently available, fluorine-18 fluorodeoxyglucose ([^18^F]F-FDG) is still the most widely used PET radiotracer in oncology [2,3,4]. [^18^F]F-FDG uptake is related to glucose metabolism, and increased glucose metabolism is one of the hallmarks of many cancer types. However, [^18^F]F-FDG has known limitations, such as its high physiological uptake in many normal tissues (hampering the detection of tumor lesions in these sites), its low uptake in certain tumor types (as several well-differentiated tumors), and a lack of specificity (as several diseases may be characterized by increasing glucose metabolism); these limitations represent the basis for the continuous development of new PET radiotracers in oncology [2,3,4].

Recently, fibroblast activation protein (FAP) expression in cancer-associated fibroblasts (CAFs) was evaluated as a possible target for PET imaging in oncology [5,6]. CAFs are the main component of tumor microenvironment, which has a pivotal role in cancer development, including tumor growth, tumor invasion and metastatic spread [7]. FAP is a transmembrane glycoprotein enzyme, which is overexpressed on the cell surface of activated CAFs of multiple tumor types and, in particular, in many epithelial carcinomas (especially in those characterized by a strong desmoplastic reaction, as they can comprise up to 90% of the tumor mass). Conversely, there is a low expression of FAP in ubiquitous resting fibroblasts of healthy tissues [7]. However, FAP expression is not cancer specific but activated fibroblasts in nonmalignant diseases may overexpress FAP [7,8].

Several radiolabeled FAP inhibitors (FAPI) targeting FAP expression in CAFs and characterized by rapid renal clearance and high tumor-to-background uptake ratio (TBR) have been developed to allow early cancer detection through PET imaging [9]. Several recent studies comparing radiolabeled FAPI and [^18^F]F-FDG as PET radiotracers in oncology have been published. The aim of this systematic review is to perform an updated evidence-based summary about the comparison of these PET radiotracers in oncology to better address further research in this setting.

## 2. Results

### 2.1. Literature Search

**Figure 1:**
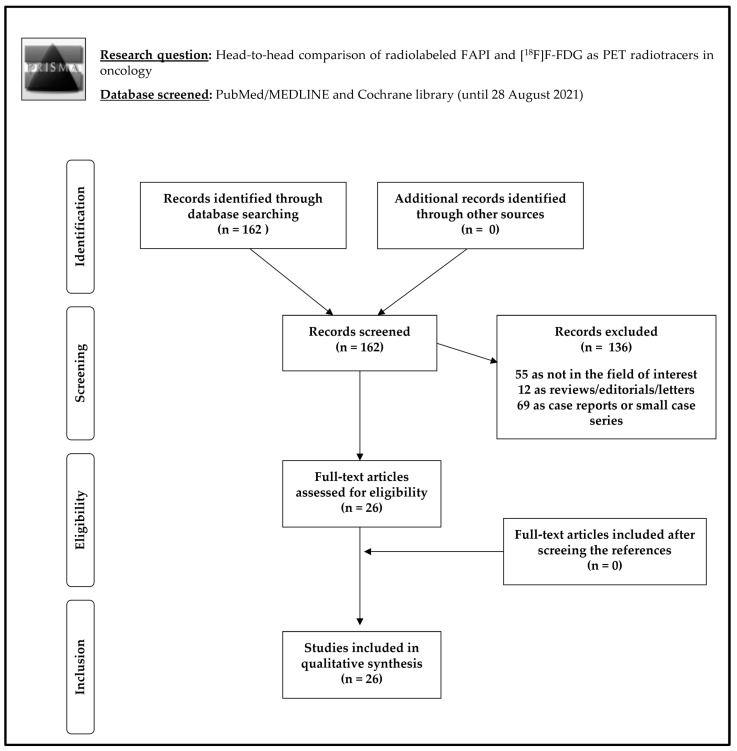
Scheme of article selection for the systematic review.

**Figure 2:**
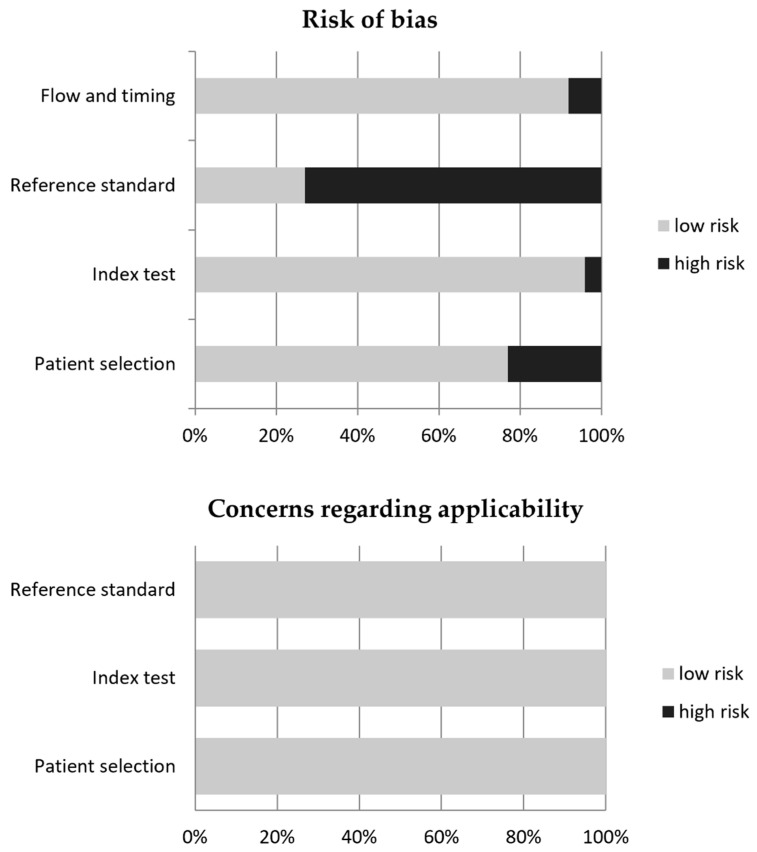
Quality assessment of the included studies according to QUADAS-2 tool.

The review question was the diagnostic comparison of radiolabeled FAPI and [^18^F]F-FDG as PET radiotracers in oncology. The literature search results using a systematic approach are reported in Figure 1. The comprehensive computer literature search from PubMed/MEDLINE and Cochrane library database revealed 162 records. Reviewing titles and abstracts, 136 records were excluded: 55 because they were not in the field of interest of this review; 12 reviews, editorials, letters or comments; and 69 case reports or small case series (< 8 patients). Twenty-six articles were selected and retrieved in full-text version. No additional studies were found screening the references of the selected articles. Finally, 26 articles (925 patients) including data on the comparison between radiolabeled FAPI and [^18^F]F-FDG as PET radiotracers in oncology were included in the systematic review [10,11,12,13,14,15,16,17,18,19,20,21,22,23,24,25,26,27,28,29,30,31,32,33,34,35]. The characteristics of the studies selected for the systematic review are presented in Table 1, Table 2, Table 3. The overall quality assessment of the studies is reported in Figure 2.

### 2.2. Qualitative Synthesis (Systematic Review)

#### 2.2.1. Basic Study and Patient Characteristics

Through the comprehensive computer literature search, 26 full-text articles including data on the head-to-head comparison of radiolabeled FAPI and [^18^F]F-FDG in cancer patients were selected (Table 1) [10,11,12,13,14,15,16,17,18,19,20,21,22,23,24,25,26,27,28,29,30,31,32,33,34,35]. All the selected articles were published in the last two years. Countries from Asia, Europe, North America and Africa were represented; the most frequent country was China followed by Germany and Turkey. About the type of study, 88% of the studies were monocentric, 12% were multicentric, 54% were retrospective and 46% were prospective. Different types of tumors were evaluated in the selected studies. The number of patients performing PET with radiolabeled FAPI and [^18^F]F-FDG ranged from 8 to 123. The median age of the patients included ranged from 44 to 70 years; the male percentage was highly variable from 0% to 96%.

#### 2.2.2. Technical Aspects

Heterogeneous technical aspects among the included studies were found (Table 2). The most frequent FAPI radiotracer used was [^68^Ga]Ga-DOTA-FAPI-04. The hybrid imaging modality was PET/CT in most of the studies; PET/MRI was also performed in 23% of included studies. The time between [^18^F]F-FDG PET and radiolabeled FAPI PET ranged from one day to 89 days, even if the most frequent time range was within one week. The radiopharmaceutical injected activity largely varied among the included studies. Notably, fasting was requested only before [^18^F]F-FDG injection, but not before radiolabeled FAPI injection. The most frequent time from the radiopharmaceutical injection to PET image acquisition was one hour for both [^18^F]F-FDG and FAPI radiotracers. The PET image analysis was performed by using qualitative (visual) analysis and additional semi-quantitative analysis through the calculation of the maximal standardized uptake values (SUVmax) in all the studies. For qualitative analysis an area of increased radiopharmaceutical uptake was considered abnormal at [^18^F]F-FDG PET and radiolabeled FAPI PET if this uptake was higher than the background region, excluding sites of physiological uptake.

#### 2.2.3. Radiotracer Biodistribution and Main Outcome Measures

Regarding the normal tissue biodistribution of radiolabeled FAPI in comparison to [^18^F]F-FDG, all the included studies showed a lower radiolabeled FAPI uptake in the normal brain, liver, and oral mucosa, compared to [^18^F]F-FDG [10,11,12,13,14,15,16,17,18,19,20,21,22,23,24,25,26,27,28,29,30,31,32,33,34,35].

The main outcome measures about the head-to-head comparison among [^18^F]F-FDG and FAPI radiotracers are listed in Table 3 and include comparison of radiopharmaceutical uptake and tumor-to-background uptake ratio (TBR) in tumor lesions, and comparison in the detection of primary tumor lesions and/or metastases.

About the comparison of the uptake of [^18^F]F-FDG and FAPI radiotracers in tumor lesions, there are discrepant findings among the included articles. A significantly higher uptake of radiolabeled FAPI, compared to [^18^F]F-FDG, was reported only in some articles and only for some types of tumors, most frequently in gastrointestinal tumors, liver tumors and breast cancer. Conversely, when investigated, most of the included articles clearly demonstrated a significant higher TBR for FAPI radiotracers, compared to [^18^F]F-FDG.

Overall, taking into account the radiotracer uptake and TBR values, compared to [^18^F]F-FDG PET, an equal or higher detection of primary tumors and/or metastatic lesions was usually demonstrated by using radiolabeled FAPI PET [10,11,12,13,14,15,16,17,18,19,20,21,22,23,24,25,26,27,28,29,30,31,32,33,34,35]. In particular, the cancer entities with better detection rate of tumor lesions by using radiolabeled FAPI PET compared to [^18^F]F-FDG PET were gastrointestinal tumors, liver tumors, breast cancer and nasopharyngeal carcinoma.

## 3. Discussion

Compared to the previous systematic reviews on FAPI imaging [8,36,37], our systematic review was focused on the head-to-head diagnostic comparison on [^18^F]F-FDG PET and radiolabeled FAPI PET in oncology, and therefore, only studies or subsets of studies performing both these imaging methods in cancer patients were selected. We believe that the head-to-head comparison should be preferred, compared to indirect comparison, to obtain more solid evidence.

Overall, we found several advantages of radiolabeled FAPI PET, compared to [^18^F]F-FDG in oncology. First of all, about the patient preparation, compared to [^18^F]F-FDG, radiolabeled FAPI PET, does not require fasting or any dietary preparation, as glucose metabolic pathways are not involved; thus, a higher patient compliance is expected, compared to [^18^F]F-FDG, as radiolabeled FAPI PET is feasible even in patients with high serum glucose levels (e.g., diabetic patients).

Most of the FAPI radiotracers included in this systematic review were labeled with ^68^Ga obtained from a ^68^Ge/^68^Ga generator; thus, the radiotracer can be produced on site also in small PET centers without an on-site cyclotron. On the other hand, the ^68^Ga activity obtained from a generator may be limited, taking into account batch size and short radionuclide half-life. Furthermore, the price of ^68^Ge/^68^Ga generators should be considered. To overcome these drawbacks, FAPI radiolabeling with the longer-lived radionuclide ^18^F was recently investigated [38]. Moreover, aside from the reduced availability of ^68^Ge/^68^Ga generators, we would like to underline that FAPI radiotracers labeled with ^68^Ga, which are the most used FAPI radiopharmaceuticals, are affected by a lower resolution for PET imaging with respect to FAPI radiotracers labeled with ^18^F, due to the high positron energy of ^68^Ga, compared to ^18^F [38].

About the normal tissue biodistribution of radiolabeled FAPI in comparison to [^18^F]F-FDG, all the included studies showed a lower radiolabeled FAPI uptake in the normal brain, liver, and oral mucosa, compared to [^18^F]F-FDG. Therefore, this is the rationale for the better detection of primary or metastatic lesions in these organs [10,11,12,13,14,15,16,17,18,19,20,21,22,23,24,25,26,27,28,29,30,31,32,33,34,35]. As radiolabeled FAPI seems to present lower background activity, compared to [^18^F]F-FDG, considering the equal or higher uptake in tumoral lesions, this may finally result in a sharper contrast [10,11,12,13,14,15,16,17,18,19,20,21,22,23,24,25,26,27,28,29,30,31,32,33,34,35]. Overall, taking into account radiotracer uptake and TBR values, compared to [^18^F]F-FDG PET, an equal or higher detection of primary tumors and/or metastatic lesions was usually demonstrated by using radiolabeled FAPI PET [10,11,12,13,14,15,16,17,18,19,20,21,22,23,24,25,26,27,28,29,30,31,32,33,34,35]. In particular, the cancer entities with better detection rate of tumor lesions by using radiolabeled FAPI PET, compared to [^18^F]F-FDG PET, were gastrointestinal tumors, liver tumors, breast cancer and nasopharyngeal carcinoma.

Furthermore, compared to [^18^F]F-FDG, using FAPI radiotracers, a theragnostic approach (e.g., diagnosis and therapy with FAPI radiotracers) seems also feasible [5].

Notably, compared to [^18^F]F-FDG, the limitation of the reduced specificity still remains with radiolabeled FAPI. As a matter of fact, [^18^F]F-FDG is known to accumulate in acute inflammation, whereas recent studies have demonstrated the increased radiolabeled FAPI uptake, due to FAP activation in chronic inflammation, causing a fibrotic reaction [8,39].

Even if the results reported by the studies included in this systematic review seem promising regarding the role of radiolabeled FAPI PET in oncology, more research studies focused on specific tumor types are still needed to clearly define the role of radiolabeled FAPI PET/CT of PET/MRI in oncology and to define whether radiolabeled FAPI may substitute [^18^F]F-FDG (e.g., in some tumor types with low glucose metabolism) or have a complementary role (e.g., possible use in patients with inconclusive findings at [^18^F]F-FDG PET).

However, the real-world scenario is still characterized by the reduced availability of radiolabeled FAPI worldwide, compared to [^18^F]F-FDG, and a small number of available research data comparing these radiotracers in specific oncological settings is currently available [39,40].

Some limitations of our systematic review should be underlined. First of all, the well-recognized clinical and methodological heterogeneity of the included studies hampered a pooled analysis (meta-analysis) and the achievement of definitive conclusions about the review question. To this regard, a meta-analysis on radiolabeled FAPI compared to [^18^F]F-FDG should be performed about specific tumor types, but unfortunately the number of articles on specific tumor types is still limited. Furthermore, some biases of the included studies should be recognized, such as a lack of adequate reference standard in some studies and the possible publication bias, particularly in studies including a low number of patients. We have tried to limit the publication bias excluding case reports and small case series from this systematic review.

Based on current literature data, we cannot still suggest the alternative or complementary use of radiolabeled FAPI PET compared to [^18^F]F-FDG PET in oncology. Further head-to-head comparison studies among radiolabeled FAPI and [^18^F]F-FDG for specific tumor types are warranted, and in particular, cost-effectiveness analyses are strongly suggested to better define the future role of radiolabeled FAPI PET in oncology, compared to [^18^F]F-FDG PET.

## 4. Materials and Methods

The reporting of this systematic review conforms to the updated “Preferred Reporting Items for a Systematic Review and Meta-Analysis” (PRISMA) statement, a reporting guidance to identify, select, appraise, and synthesize studies in systematic reviews [41].

### 4.1. Search Strategy

Two authors (G.T. and B.M.) independently performed a comprehensive computer literature search of PubMed/MEDLINE and Cochrane library databases to find relevant articles comparing radiolabeled FAPI and [^18^F]F-FDG as PET radiotracers in oncology.

A search algorithm based on a combination of these terms was used: ((FDG) OR (fluorodeoxyglucose)) AND ((FAPI) OR (FAP) OR (fibroblast activation protein)). No beginning date limit was used. The search was updated until 28 August 2021. No language restriction was used. To expand the search, references of the retrieved articles were also screened for additional studies.

### 4.2. Study Selection

Studies or subsets of studies comparing radiolabeled FAPI and [^18^F]F-FDG as PET radiotracers in oncology were eligible for inclusion in the systematic review. The exclusion criteria were (a) articles not within the field of interest of this review, including studies not comparing these radiopharmaceuticals or those comparing them, but in other field than in oncology; (b) review articles, editorials, letters, comments, conference proceedings related to the review question; and (c) case reports or small case series related to the review question (<8 patients).

Two researchers (G.T. and B.M.) independently reviewed the titles and abstracts of the retrieved articles, applying the inclusion and exclusion criteria mentioned above. Articles were rejected if they were clearly ineligible. The same two researchers then independently reviewed the full-text version of the remaining articles to assess their eligibility for inclusion. Disagreements were resolved in an online consensus meeting involving all the co-authors.

### 4.3. Data Extraction

For each included study, information was collected by two authors independently (G.T. and B.M.) concerning basic study (authors, year of publication, country of origin, study design), patient characteristics (type or cancer evaluated, number of patients who underwent PET with both radiotracers, mean/median age, sex ratio), technical aspects (type of radiotracers, PET hybrid imaging modality and tomographs, time between PET with radiolabeled FAPI and [^18^F]F-FDG, radiotracer injected activity, time interval between radiotracer injection and image acquisition, image analysis and reference standard). Furthermore, main findings of the included studies about the comparison among [^18^F]F-FDG and FAPI radiotracers were extracted. In particular, the results on the comparison of radiopharmaceutical uptake, tumor-to-background uptake ratio (TBR) in tumor lesions, and detection of primary tumor lesions and/or metastases were extracted from the original studies.

### 4.4. Quality Assessment

The overall quality of the studies included in the systematic review was critically appraised by two authors (G.T. and B.M.) based on the revised “Quality Assessment of Diagnostic Accuracy Studies” tool (QUADAS-2) [42].

### 4.5. Statistical Analysis

Due to the significant methodological and clinical heterogeneity (considering the different types of tumors evaluated) a statistical analysis was not performed to avoid additional statistical heterogeneity [40,43,44].

## 5. Conclusions

Literature data about the comparison of [^18^F]F-FDG and radiolabeled FAPI as PET radiotracers in oncology are rapidly increasing. Overall, taking into account radiotracer uptake and TBR values, compared to [^18^F]F-FDG PET, an equal or higher detection of primary tumors and/or metastatic lesions was usually demonstrated by using radiolabeled FAPI PET. In particular, the cancer entities with better detection rate of tumor lesions by using radiolabeled FAPI PET compared to [^18^F]F-FDG PET were gastrointestinal tumors, liver tumors, breast cancer and nasopharyngeal carcinoma. Further comparison studies are inevitably needed to better evaluate the best field of application of each PET radiotracer.

## Figures and Tables

**Table 1:** Basic study and patient characteristics of the included studies.

Authors	Year	Type of Study	Country	Cancer Evaluated	PET Radiopharmaceuticals	No. of Cases Compared	Age (Years)	Male%
Ballal et al. [10]	2021	P-Mo	India	Various cancers	[^18^F]F-FDG and [^68^Ga]Ga-DOTA.SA.FAPI	54	48.4 (mean)	37%
Chen et al. [11]	2020	P-Mo	China	Various cancers	[^18^F]F-FDG and [^68^Ga]Ga-DOTA-FAPI-04	75	61.5 (median)	63%
Chen et al. [12]	2021	P-Mo	China	Various cancers	[^18^F]F-FDG and [^68^Ga]Ga-DOTA-FAPI-04	68	57 (median)	59%
Dendl et al. [13]	2021	R-Bi	Germany and South Africa	Gynecological cancers	[^18^F]F-FDG and [^68^Ga]Ga-DOTA-FAPI tracers (FAPI-02, FAPI-04 or FAPI-46)	10	59.5 (median)	0%
Elboga et al. [14]	2021	R-Mo	Turkey	Breast cancer	[^18^F]F-FDG and [^68^Ga]Ga-DOTA-FAPI-04	48	53.3 (mean)	0%
Giesel et al. [15]	2021	R-Mu	Germany, USA and South Africa	Various cancers	[^18^F]F-FDG and [^68^Ga]Ga-DOTA-FAPI tracers (FAPI-02, FAPI-04, FAPI-46 or FAPI-74)	71	60 (median)	61%
Guo et al. [16]	2021	R-Mo	China	Liver cancer	[^18^F]F-FDG and [^68^Ga]Ga-DOTA-FAPI-04	34	60.6 (mean)	74%
Jiang et al. [17]	2021	R-Bi	China	Gastric cancer	[^18^F]F-FDG and [^68^Ga]Ga-DOTA-FAPI-04	38	67.5 (median)	76%
Kessler et al. [18]	2021	P-Mo	Germany	Sarcoma	[^18^F]F-FDG and [^68^Ga]Ga-DOTA-FAPI-46	43	48.1 (mean)	51%
Kömek et al. [19]	2021	P-Mo	Turkey	Breast cancer	[^18^F]F-FDG and [^68^Ga]Ga-DOTA-FAPI-04	20	44 (median)	0%
Kreppel et al. [20]	2021	R-Mo	Germany	Liver metastases of NETs	[^18^F]F-FDG, [^68^Ga]Ga-DATA5m.SA.FAPI and [^68^Ga]Ga-DOTA-TOC	13	66.8 (mean)	62%
Kuten et al. [21]	2021	P-Mo	Israel	Gastric cancer	[^18^F]F-FDG and [^68^Ga]Ga-DOTA-FAPI-04	13	70 (median)	46%
Lan et al. [22]	2021	P-Mo	China	Various cancers	[^18^F]F-FDG and [^68^Ga]Ga-DOTA-FAPI-04	123	56.1 (mean)	56%
Linz et al. [23]	2021	P-Mo	Germany	Oral cancer	[^18^F]F-FDG and [^68^Ga]Ga-DOTA-FAPI-04	10	62 (mean)	80%
Pang et al. [24]	2021	R-Mo	China	Gastrointestinal cancers	[^18^F]F-FDG and [^68^Ga]Ga-DOTA-FAPI-04	35	64 (median)	72%
Qin et al. [25]	2021	P-Mo	China	Gastric cancer	[^18^F]F-FDG and [^68^Ga]Ga-DOTA-FAPI-04	20	56 (median)	45%
Qin et al. [26]	2021	P-Mo	China	Nasopharyngeal cancer	[^18^F]F-FDG and [^68^Ga]Ga-DOTA-FAPI-04	15	51.2 (mean)	53%
Qin et al. [27]	2021	R-Mo	China	Bone metastases or bone and joint lesions	[^18^F]F-FDG and [^68^Ga]Ga-DOTA-FAPI-04	29	56.6 (mean)	57%
Sahin et al. [28]	2021	R-Mo	Turkey	Liver metastases of gastrointestinal cancers	[^18^F]F-FDG and [^68^Ga]Ga-DOTA-FAPI-04	31	61.9 (mean)	61%
Serfling et al. [29]	2021	R-Mo	Germany	Suspicious tonsillary tumor or CUP	[^18^F]F-FDG and [^68^Ga]Ga-DOTA-FAPI-04	8	62 (mean)	75%
Shi et al. [30]	2021	P-Mo	China	Liver cancer	[^18^F]F-FDG and [^68^Ga]Ga-DOTA-FAPI-04	20	58 (mean)	90%
Wang et al. [31]	2021	P-Mo	China	Various cancers	[^18^F]F-FDG and Al [^18^F]F-NOTA-FAPI	10	63.6 (mean)	40%
Wang et al. [32]	2021	R-Mo	China	Liver cancer	[^18^F]F-FDG and [^68^Ga]Ga-DOTA-FAPI-04	25	59.4 (mean)	96%
Zhao et al. [33]	2021	R-Mo	China	Esophageal cancer	[^18^F]F-FDG and [^68^Ga]Ga-DOTA-FAPI-04	21	60 (median)	86%
Zhao et al. [34]	2021	R-Mo	China	Peritoneal carcinomatosis	[^18^F]F-FDG and [^68^Ga]Ga-DOTA-FAPI-04	46	57 (median)	30%
Zhao et al. [35]	2021	R-Mo	China	Nasopharyngeal cancer	[^18^F]F-FDG and [^68^Ga]Ga-DOTA-FAPI-04	45	50 (median)	78%

Legend: [^18^F]F = fluorine-18; [^68^Ga]Ga = gallium-68; Bi = bicentric; CUP = cancer of unknown primary; DOTA = dodecane tetraacetic acid; FAPI = fibroblast activation protein inhibitor; FDG = fluorodeoxyglucose; male% = male percentage; Mo = monocentric; Mu = multicentric; NETs = neuroendocrine tumors; P = prospective; PET = positron emission tomography; R = retrospective.

**Table 2:** Technical aspects of the included studies.

Authors	PET Hybrid Modality and Tomograph	Time between [^18^F]F-FDG and Radiolabeled FAPI PET	Mean [^18^F]F-FDG Injected Activity	Time between [^18^F]F-FDG injection and PET Acquisition	Mean Radiolabeled FAPI Injected Activity	Time between Radiolabeled FAPI Injection and PET Acquisition	PET Image Analysis	Reference Standard
Ballal et al. [10]	GE Discovery 710 PET/CT	within one week	271 MBq	1 h	144.3 MBq	1 h	Q and SQ	Composite
Chen et al. [11]	GE Discovery MI PET/CT	within one week	3.7 MBq/kg	1 h	1.8–2.2 MBq/kg	1 h	Q and SQ	Histology
Chen et al. [12]	GE Discovery MI PET/CT	within one week	3.7 MBq/kg	1 h	1.8–2.2 MBq/kg	1 h	Q and SQ	Composite
Dendl et al. [13]	Siemens Biograph mCT PET/CT	1–89 days	304 MBq	1 h	185 MBq	1 h	Q and SQ	Composite
Elboga et al. [14]	GE Discovery IQ PET/CT	within one week	3.5–5.5 MBq/kg	1 h	2 MBq/kg	1 h	Q and SQ	Composite
Giesel et al. [15]	Siemens Biograph mCT or GE Discovery IQ PET/CT	1–89 days	316 MBq	1 h	185 MBq	1 h	Q and SQ	Composite
Guo et al. [16]	GE Discovery MI PET/CT	within one week	3.7 MBq/kg	1 h	148–259 MBq	1 h	Q and SQ	Composite
Jiang et al. [17]	United Imaging uPMR790 TOF PET/MRI; Siemens Biograph mCT, Philips Ingenuity TF or United Imaging uMI510 PET/MRI	NR	NR	1 h	111–185 MBq	1 h	Q and SQ	Histology
Kessler et al. [18]	Siemens Biograph mMR PET/MRI; Siemens Biograph mCT PET/CT	within four weeks	214 MBq	1 h	144 MBq	10 min	Q and SQ	Histology
Kömek et al. [19]	GE Discovery IQ PET/CT	within one week	3.5–5.5 MBq/kg	1 h	2 MBq/kg	1 h	Q and SQ	Composite
Kreppel et al. [20]	Siemens Biograph 2, Philips Gemini GXL, or GE Discovery STE PET/CT	NR	267 MBq	74 min	184 MBq	79 min	Q and SQ	Histology
Kuten et al. [21]	GE Discovery MI PET/CT	1–23 days	3.7 MBq/kg	1 h	1.8–2.2 MBq/kg	1 h	Q and SQ	Composite
Lan et al. [22]	United Imaging uMI780 PET/CT	within three days	3.7 MBq/kg	45–60 min	1.85 MBq/kg	1 h	Q and SQ	Composite
Linz et al. [23]	Siemens Biograph mCT PET/CT	2–16 days	269 MBq	1 h	119 MBq	1 h	Q and SQ	Histology
Pang et al. [24]	GE Discovery MI PET/CT	within one week	3.7 MBq/kg	1 h	1.8–2.2 MBq/kg	1 h	Q and SQ	Histology
Qin et al. [25]	GE SIGNA PET/MRI; GE Discovery VCT PET/CT	within one week	3.7–5.55 MBq/kg	1 h	1.85–3.7 MBq/kg	30–60 min	Q and SQ	Composite
Qin et al. [26]	GE SIGNA PET/MRI; GE Discovery VCT PET/CT	within one week	3.7–5.4 MBq/kg	1 h	1.85–3.7 MBq/kg	30–60 min	Q and SQ	Composite
Qin et al. [27]	GE SIGNA PET/MRI; GE Discovery VCT PET/CT	within one week	NR	NR	1.85–3.7 MBq/kg	20–60 min	Q and SQ	Composite
Sahin et al. [28]	GE Discovery IQ PET/CT	at least two weeks	5 MBq/kg	1 h	2–3 MBq/kg	45 min	Q and SQ	Composite
Serfling et al. [29]	Siemens Biograph mCT PET/CT	within one week	292 MBq	1 h	145 MBq	1 h	Q and SQ	Histology
Shi et al. [30]	Sinounion Healthcare PoleStar m660 PET/CT	within three days	3.7 MBq/kg	60–90 min	3.59 MBq/kg	40–50 min	Q and SQ	Composite
Wang et al. [31]	Siemens Biograph mCT PET/CT	NR	NR	NR	173.5–256.8 MBq	60–90 min	Q and SQ	Composite
Wang et al. [32]	Siemens Biograph mCT or Union Imaging uMI510 PET/CT	within one day	NR	NR	185 MBq	1 h	Q and SQ	Composite
Zhao et al. [33]	GE Discovery MI PET/CT	within one week	3.7–5.5 MBq/kg	1 h	1.8–2.2 MBq/kg	1 h	Q and SQ	Composite
Zhao et al. [34]	GE Discovery MI PET/CT	within one week	3.7 MBq/kg	1 h	1.8–2.2 MBq/kg	1 h	Q and SQ	Composite
Zhao et al. [35]	GE Discovery MI PET/CT	NR	3.7 MBq/kg	40 min	1.8–2.2 MBq/kg	40 min	Q and SQ	Composite

Legend: [^18^F]F = fluorine-18; Composite = histology + imaging/clinical/laboratory follow-up; CT = computed tomography; FAPI = fibroblast activation protein inhibitor; FDG = fluorodeoxyglucose; h = hour; kg = kilograms; MBq = megabecquerel; min = minutes; MRI = magnetic resonance imaging; NR = not reported; PET = positron emission Tomography; Q = qualitative; SQ = semiquantitative.

**Table 3:** Main results of the included studies about the comparison among [^18^F]F-FDG and FAPI radiotracers.

Authors	Cancer Evaluated	Significant Higher Uptake of Radiolabeled FAPI Compared to [^18^F]F-FDG	Significant Higher TBR of Radiolabeled FAPI Compared to [^18^F]F-FDG	Comparison in the Detection of Primary Tumors	Comparison in the Detection of Metastases
Ballal et al. [10]	Various cancers	only for brain metastases	only for brain metastases	NR	NR
Chen et al. [11]	Various cancers	yes	yes	FAPI > FDG	FAPI > FDG
Chen et al. [12]	Various cancers	yes	yes	FAPI > FDG	FAPI > FDG
Dendl et al. [13]	Gynecological cancers	no	only for distant metastases	NR	NR
Elboga et al. [14]	Breast cancer	yes	NR	FAPI > FDG	FAPI > FDG
Giesel et al. [15]	Various cancers	no	only for liver and bone metastases	NR	NR
Guo et al. [16]	Liver cancer	yes	yes	FAPI > FDG	FAPI > FDG
Jiang et al. [17]	Gastric cancer	no	yes	FAPI > FDG	FAPI = FDG
Kessler et al. [18]	Sarcoma	no	yes	FAPI = FDG	FAPI = FDG
Kömek et al. [19]	Breast cancer	yes	yes	FAPI > FDG	FAPI > FDG
Kreppel et al. [20]	Liver metastases of NETs	yes	NR	NR	FAPI > FDG
Kuten et al. [21]	Gastric cancer	no	yes	FAPI > FDG	FAPI > FDG
Lan et al. [22]	Various cancers	yes	no	FAPI > FDG	FAPI > FDG
Linz et al. [23]	Oral cancer	no	NR	FAPI = FDG	FAPI = FDG
Pang et al. [24]	Gastrointestinal cancers	yes	NR	FAPI > FDG	FAPI > FDG
Qin et al. [25]	Gastric cancer	yes	yes	FAPI > FDG	FAPI > FDG
Qin et al. [26]	Nasopharyngeal cancer	no	NR	FAPI = FDG	FAPI > FDG
Qin et al. [27]	Bone metastases or bone and joint lesions	no	NR	NR	FAPI > FDG
Sahin et al. [28]	Liver metastases of gastrointestinal cancers	no	yes	NR	FAPI > FDG
Serfling et al. [29]	Suspicious tonsillary tumor or CUP	no	yes	FAPI = FDG	FAPI < FDG
Shi et al. [30]	Liver cancer	yes	yes	FAPI > FDG	FAPI > FDG
Wang et al. [31]	Various cancers	no	yes	FAPI = FDG	FAPI > FDG
Wang et al. [32]	Liver cancer	no	yes	FAPI > FDG	FAPI > FDG
Zhao et al. [33]	Esophageal cancer	yes	NR	NR	NR
Zhao et al. [34]	Peritoneal carcinomatosis	yes	NR	NR	FAPI > FDG
Zhao et al. [35]	Nasopharyngeal cancer	yes	NR	FAPI = FDG	FAPI > FDG

Legend: [^18^F]F = fluorine-18; CT = computed tomography; FAPI = fibroblast activation protein inhibitor; FDG = fluorodeoxyglucose; NR = not reported; PET = positron emission tomography; TBR = tumor-to-background ratio.

## Data Availability

Data used in this review article were extracted from scientific articles listed in PubMed/MEDLINE and Cochrane Library databases.
